# Plant Functional Traits Are the Mediators in Regulating Effects of Abiotic Site Conditions on Aboveground Carbon Stock-Evidence From a 30 ha Tropical Forest Plot

**DOI:** 10.3389/fpls.2018.01958

**Published:** 2019-01-09

**Authors:** Wensheng Bu, Jihong Huang, Han Xu, Runguo Zang, Yi Ding, Yide Li, Mingxian Lin, Jinsong Wang, Cancan Zhang

**Affiliations:** ^1^2011 Collaborative Innovation Center of Jiangxi Typical Trees Cultivation and Utilization, Jiulianshan National Observation and Research Station of Chinese Forest Ecosystem, College of Forestry, Jiangxi Agricultural University, Nanchang, China; ^2^Key Laboratory of Forest Ecology and Environment of State Forestry Administration, Institute of Forest Ecology, Environment and Protection, Chinese Academy of Forestry, Beijing, China; ^3^Co-Innovation Center for Sustainable Forestry in Southern China, Nanjing Forestry University, Nanjing, China; ^4^Key Laboratory of State Forestry Administration on Tropical Forestry Research, Research Institute of Tropical Forestry, Chinese Academy of Forestry, Guangzhou, China; ^5^Key Laboratory of Ecosystem Network Observation and Modeling, Institute of Geographic Sciences and Natural Resources Research, Chinese Academy of Sciences, Beijing, China

**Keywords:** biodiversity and ecosystem function, plant economics spectrum, response-effect framework, specific leaf area, wood density

## Abstract

Understanding the relative contribution of abiotic and biotic factors to the formation of ecosystem functioning across scales is vital to evaluate ecosystem services. Here, we elucidate the effects of abiotic site conditions (i.e., soil and topographic properties) and plant functional traits on variations of stand aboveground carbon (AGC) stock in an old-growth tropical montane rain forest. The response-effect framework in functional ecology is adopted in examining how plant functional traits respond to environmental changes and affect ecosystem functioning. We measured specific leaf area and wood density of 270 woody plant species and estimated stand AGC stocks in a 30-ha forest plot. The relationships among environmental factors (ENVIRONMENT), community-weighted means of functional traits (TRAITS) and stand AGC stocks across nested spatial scales were disentangled by structural equation modeling. The results showed that the stands composed of ‘acquisitive’ species (high specific leaf area and low wood density) had low AGC, whereas stands composed of ‘conservative’ species (low specific leaf area and high wood density) had high AGC. TRAITS responded to ENVIRONMENT and affected AGC directly. ENVIRONMENT had an indirect effect on AGC through its direct effect on TRAITS. TRAITS were more important than ENVIRONMENT in driving variations of AGC. The effects of TRAITS on AGC increased, while the effects of ENVIRONMENT on AGC decreased with the increase of spatial scales in the tropical montane rain forest. Our study suggests that plant functional traits are the mediators in regulating effects of abiotic site conditions on ecosystem functions.

## Introduction

A variety of global changes are driving an increase in rates of species extinctions. Biodiversity loss in the 21st century has been one of the major drivers of ecosystem change (Hooper et al., 2012). Over 60% of ecosystem services are deteriorating or are already overused at global scale (Assessment, 2005), understanding the underlying mechanisms of biotic communities in regulating ecosystem processes is a pressing issue in ecology (Liu et al., 2018). Many studies have proved that ecosystem functions are affected by species composition (Liu et al., 2018), functional trait composition of the community (Liu et al., 2016); trait diversity and phylogenetic diversity (Cadotte et al., 2009) and tree size (Stephenson et al., 2014) and so on.

Functional traits are defined as morpho-physio-phenological traits that impact the fitness of individual species via their effects on growth, reproduction and survival, the three components of individual performance (Violle et al., 2007). Moreover, functional traits can indicate how a species relates and responds to its environment, which offers a powerful approach to addressing ecological questions (McGill et al., 2006). Abiotic site conditions (i.e., soil, topographic properties and light environment) can influence ecosystem processes directly by changing the ecosystem flux rate of energy and matter or indirectly by changing the physiological rate of plants, which is determined by the functional traits of plants (Boyer et al., 2009; Geert Hiddink et al., 2009; Lavorel et al., 2011; Hajek et al., 2016). As for plant functions, at least two dimensions exist, although it could be extended to include more dimensions (Westoby et al., 2002). Two of these dimensions were leaf economics spectrum (LES) and wood economics spectrum (WES). LES and WES are most commonly represented by specific leaf area (Wright et al., 2004) and wood density (Chave et al., 2009), respectively. These economics spectra can respond to environmental conditions, e.g., leaf traits are influenced by soil resource availability (Ordoñez et al., 2009) and topographical factors (Butterfield and Suding, 2013), while wood density is affected by soil fertility (Nishimua et al., 2007). Furthermore, these economics spectra also affect ecosystem functions, e.g., LES is related to biogeochemistry (Freschet et al., 2010) and above-ground net primary productivity (Mokany et al., 2008) while wood density is correlated with stem growth rate and carbon stock (Chao et al., 2008). Therefore, understanding the effects of environmental drivers on ecosystem functions through plant functional composition is central to predicting the net provisioning of multiple ecosystem services (Lavorel and Grigulis, 2012).

There is a growing consensus that community functional composition determines ecosystem functioning and related services (Garnier et al., 2004; Petchey and Gaston, 2006; Díaz et al., 2007; Lavorel, 2013). There are two hypotheses to explain how species traits within a community may influence ecosystem processes. The mass ratio hypothesis suggests that the effect of a given species on ecosystem processes is proportional to its relative contribution to the total biomass of the community (additive effect) (Grime, 1998). While the functional diversity hypothesis implies that the effects of species on ecosystem processes are not predictable from single-species traits due to antagonistic or synergistic interactions among species (Petchey et al., 2004; Mouillot et al., 2011). These non-additive effects have been considered to be mainly caused by complementary use of resources and facilitation or competition (Hooper et al., 2005; Dias et al., 2013). Several studies suggested that plant functional trait effects on ecosystem functions were mainly due to the biomass ratio mechanism (Mokany et al., 2008; Laughlin, 2011; Lavorel et al., 2011).

Global climatic change as a result of rising level of carbon dioxide in the atmosphere has greatly stimulated the interest in biological carbon sequestration in natural ecosystems, such as forests. Connecting with a series of ecosystem processes, forest carbon stock is considered as one of the paramount indicators of forest ecosystem functioning. An effective climate control by terrestrial ecosystems not only depends on the rate of carbon uptake by primary producers, but also on the rate of carbon release from the biota (Díaz et al., 2009). Carbon stocks at any given point in time reflect the net balance between these uptake, loss and retention processes. As different plant species differ in their ability to capture, store and release carbon, the functional composition of plant communities, under a given regional climatic regime, should be a major driver of carbon sequestration in terrestrial ecosystems (De Deyn et al., 2008).

Recent ecosystem research tried to incorporate functional traits into a response-effect framework, which examines the response of plants to environmental change by the “response traits” and plant effect on ecosystem functions by the “effect traits” (Grace, 2006; Suding et al., 2008). Structural equation modeling (SEM) enables scientists to use field data to test hypotheses about causal relations and pathways of ecosystem functioning (Shipley, 2002). As the response-effect framework has the potential to incorporate community level functional traits into the impacts of environmental conditions on ecosystem functioning, disentangling the complex interactions among environment, traits and ecosystem functions, it is well suited for the analysis of SEM.

Most of the studies have just focused on the relationships between environmental conditions and plant functional traits or on the relations between functional traits and ecosystem functions. A few studies have integrated environment, traits and ecosystem functions into one system to explore the interactions among these three aspects (Lavorel and Grigulis, 2012; Liu et al., 2016). For an example, a study has shown that individual-level functional traits associated with soil environment and identity of neighboring individuals strongly predict ecosystem functions (e.g., tree growth rate) in a subtropical forest (Liu et al., 2016). Therefore, knowledge about linkage between environmental factors and ecosystem functioning through functional traits in natural ecosystems is still lacking. In this paper, we assess the relative effects of environmental variations and community functional composition on aboveground carbon (AGC) stock in a 30-ha old-growth tropical montane rainforest plot on Hainan Island, China. Using the response-effect framework of Suding et al. (2008), we built a SEM to elucidate how environmental factors influence AGC stock through plant functional traits across three nested spatial scales (40 m × 40 m, 60 m × 60 m, and 80 m × 80 m) in this forest plot. We hypothesized that: (1) Both abiotic (soil/topographic) factors and biotic factors (plant functional traits) had significant influences on the stand AGC stock at each spatial scale. (2) Plant functional traits at community level mediate the links between physical environmental variations and stand AGC stock through the response-effect framework. (3) Plant functional trait effects on stand AGC stock should increase with increasing spatial scale due to additive effects, meanwhile environmental effects on stand AGC stock should decrease with increasing spatial scale due to the homogenization of environmental variables.

## Materials and Methods

### Study Site

This study was conducted in a 30-ha (500 m × 600 m) forest plot of old-growth (naturally grows over 150 years) tropical montane rainforest in Jianfengling Nature Reserve (18°23^′^–18°50^′^N, 108°36^′^–109°05^′^E) on Hainan Island in southern China. The Jianfengling Nature Reserve is located at the northern edge of the Asian tropical forest, covering ca 640 km^2^, with an elevation range of ca 0–1,412 m a.s.l. The mean annual temperature is 19.7°C and annual precipitation is 2,651 mm, with a distinct wet season from May to October and a dry season from November to April of the following year. The soil type is latosol, which is broadly distributed in tropical regions of south China (Jiang et al., 2002).

### Data Collection

Field investigation was conducted in the 30-ha plot of old-growth tropical montane rainforests and data collection followed the plot census protocol established by the Center for Tropical Forest Sciences Network (John et al., 2007). The entire 500 m × 600 m plot was divided into 750 quadrats of 20 m × 20 m and elevations at the four corners of each quadrat were measured by the total station (Topcon GTS332N total station, Topcon, Japan). The study area is characterized by a rugged terrain (elevations ranged from 868.3 to 975.2 m and slopes in the 20 m × 20 m quadrat ranged from 1.7° to 49.2°). All plants with diameter at breast height (DBH) ≥ 1 cm were tagged, identified, measured and mapped in the summer of 2011.

For soil sampling, 195 samples that come from the center of every 40 m × 40 m quadrat were arrayed throughout the plot in a regular grid and referred to as “base points.” For every “base point,” two additional sampling points were located nearby. Each additional point was located at a random compass bearing away from its associated base point. One-third (1/3) of the randomly selected additional points were located at 2 m away from their associated base points, one third (1/3) were located at 5 m away, and one third (1/3) were located at 15 m away. A soil core with 4 cm diameter and 10 cm depth was collected in each sampling point in 2012 and a total of 585 soil samples were sampled in this 30-ha plot. Soil organic matter (SOM), pH, total and available nitrogen, phosphorus, and potassium, exchangeable potassium, exchangeable sodium, exchangeable calcium, exchangeable magnesium, and exchangeable base (EB) concentrations were determined according to Forest Soil Analysis Methods (Zhang et al., 1999).

A total of 270 tree and shrub species (belonging to 145 genera and 71 families) was found in the 30-ha plot. The leaf traits were measured for all these species in at least 10 well-growth individuals per species (4,203 individuals in total) and wood density was measured for at least five individuals per species (2,015 individuals in total) in the plot or nearby the plot from July to October (growing season) in 2011, using standardized protocols for plant functional trait measurements (Cornelissen et al., 2003). For each individual, two to ten recently expanded leaves, including petioles and rachises of compound leaves were collected. Leaf surface area was measured with a leaf area meter (LI-COR 3100C Area Meter, LI-COR, Lincoln, NE, United States). Laminar dry mass was measured by drying the specimen to a constant mass at 60°C (around 72 h) and specific leaf area (SLA, cm^2^ g^-1^) was calculated for each lamina as the ratio of leaf surface area to leaf mass. Wood density was determined from increment cores and trunks. The cores were taken from bark to pith of trees (DBH ≥ 5 cm) with an increment borer. Core lengths were then measured with a Mitutoyo digital micrometer and core volumes computed for cylinders of the measured length and inner diameter of the borer. For some shrub species, we had to sample trunk for individuals (DBH ≤ 5 cm) nearby the plot and removed the phloem and bark, measured fresh volume by water displacement. Each sample was quickly wrapped in a sealed plastic bag after sampling. These samples were drying in an oven at 103°C for over 72 h until they became constant weight. Wood density (WD, g cm^-3^) was computed as the ratio of oven dry mass to fresh volume and averaged for each species.

We partitioned the 30-ha plot into three spatial scales: 40 m × 40 m (180 subplots), 60 m × 60 m (80 subplots), 80 m × 80 m (42 subplots) and measured the abundance weighted community mean trait for specific leaf area (CWM_SLA) and wood density (CWM_WD) in each scale. CWM trait values were calculated as the mean across species of their trait value weighted by the species relative abundance (Ackerly and Cornwell, 2007) using the FD package in R 2.14 (R Development Core Team, 2011) with the function dbFD (Villéger et al., 2008).

We calculated stand aboveground biomass using the following allometric regression for moist forest (Chave et al., 2014): AGB = 0.0559 ρ(DBH)^2^H, where ρ is the wood density for each species (g cm^-3^), DBH is the diameter at breast height (1.30 m in cm) and H is tree height (m). We converted plant woody biomass to carbon stock multiplying it by a factor of 0.5, since carbon stock roughly represents 50% of dry woody biomass (Tomlinson et al., 2013). We focused on AGC stock because carbon stocks at any given point in time reflect the net balance between uptake, loss and retention processes (Conti and Díaz, 2013).

### Statistical Analysis

**FIGURE 1:**
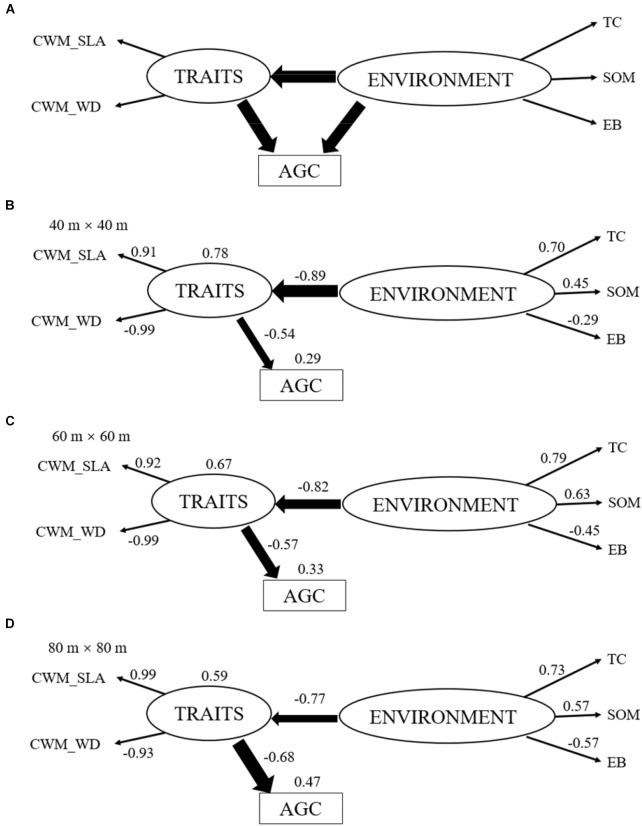
Hypothesized structural equation model **(A)**. This model provides a multivariate perspective about how abiotic site conditions [ENVIRONMENT, including terrain convexity (TC), soil organic matter (SOM) and exchangeable base (EB)] and functional traits at community level [TRAITS, including specific leaf area and wood density at community level (CWM_SLA and CWM_WD)] interact to influence aboveground carbon stock (AGC). One-headed arrows represent the hypothesized causal relationships between variables. The final structural equation models at 40 m × 40 m scale **(B)**, 60 m × 60 m scale **(C)**, 80 m × 80 m scale **(D)**. The value nearby arrows represents for the standardized regression path coefficient (*P* < 0.05) and values at the upper right corner of variables represent for the percentage of variance explained by the model. Arrow width indicates the strength of the relationship. The paths of ENVIRONMENT to AGC at these three scales were deleted, due to the non-significant relationship (*P* > 0.05).

To meet model assumptions, some predictors (SOM, EB, SLA, AGC) were a log-transformed. To assess how community weighted means of functional traits affect AGC, we examined the relationships between CWM_SLA/CWM_WD and AGC in each spatial scale by linear regression. Based on the response-and-effect framework (see Figure 1a in Suding et al., 2008), to explore the causal relationship among abiotic environmental factors, functional traits, and AGC, we used SEMs to estimate the path coefficients and variation of dependent variables. We hypothesized that abiotic environmental factors affect the functional trait and AGC, respectively, and functional traits will also directly affect AGC. In other words, we expected that abiotic environment affect AGC indirectly via their effect on plant traits. To reduce the complexity of the SEM, we projected TRAITS and ENVIRONMENT in a redundancy analysis (RDA) axis at each spatial scale (40 m × 40 m, 60 m × 60 m and 80 m × 80 m) and chose the most relevant variables to reduce redundancy among these explanatory variables (see Supplementary Figure S1). After this procedure, the parameters retained for ENVIRONMENT were terrain convexity (TC), SOM, and EB, since TC and SOM were tightly associated with CWM_WD, as well as EB was tightly associated with CWM_SLA at each spatial scale. Thus, we assumed that functional traits (TRAITS) is a latent variable, which is related to the real measured variables (CWM_SLA and CWM_WD, the typical indicators of LES and WES); Environmental factors (ENVIRONMENT) is also a latent variable, which is related to the real measured variables (TC, SOM, and EB). The final hypothesized SEM (Figure 1A) will run at different spatial scales and all SEMs was fit using maximum likelihood as implemented in Amos 18.0.1 software (Amos Development Corp., Spring House, PA, United States). The quality of SEM was judged by the parameters of model (*χ*^2^, *CMIN/df, CFI, RMSEA* and *P-*value). The direct effects were showed in the final SEMs, meanwhile the indirect effects occur if two variables are connected through paths to and from a third variable.

## Results

### The Influence of Community-Weighted Means of Functional Traits on Stand Aboveground Carbon Stocks at Various Spatial Scales

**FIGURE 2:**
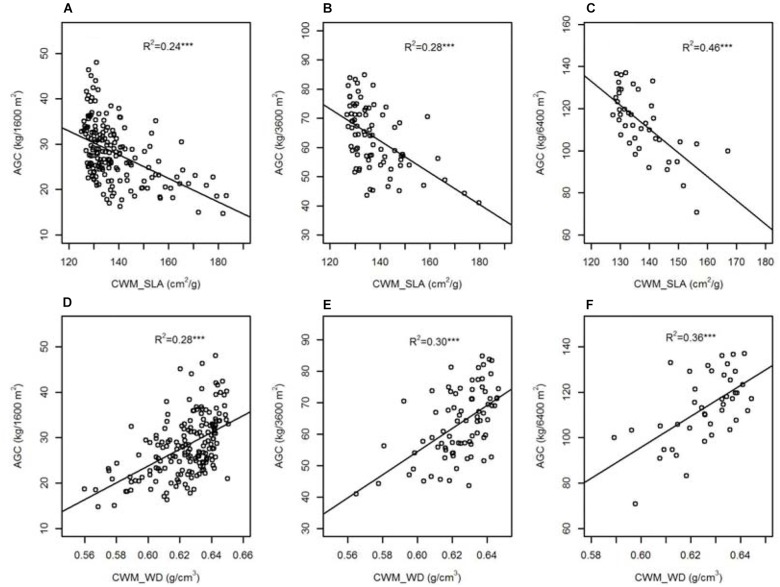
The relationships between specific leaf area/wood density at community level (CWM_SLA/CWM_WD) and aboveground carbon stock (AGC) across nested spatial scale [**(A,D)** 40 m × 40 m, **(B,E)** 60 m × 60 m, **(C,F)** 80 m × 80 m]. ^∗∗∗^*P* < 0.001.

Results of linear regressions (Figure 2) indicated that functional traits at community level showed significant linear relations with AGC in each spatial scale. Specific leaf area at community level (CWM_SLA) was negatively associated with AGC and the effect of CWM_SLA on AGC increased with increasing spatial scale (*R*^2^ from 0.24 to 0.46). However, wood density at community level (CWM_WD) was positively associated with AGC and the effect of CWM_WD on AGC increased with increasing spatial scale (*R*^2^ from 0.28 to 0.36).

### The Relationships Among Environmental Factors, Functional Traits, and Carbon Stocks at Various Spatial Scales

The final SEMs showed good consistency with the field data at 40 m × 40 m (*χ*^2^ = 14.859*, df* = 8*, n* = 180*, P* = 0.062, *CMIN/df* = 1.857, *CFI* = 0.986, *RMSEA* = 0.069), at 60 m × 60 m (*χ*^2^ = 14.894, *df* = 8, *n* = 80, *P* = 0.061, *CMIN/df* = 1.862, *CFI* = 0.973, *RMSEA* = 0.104) and at 80 m × 80 m (*χ*^2^ = 10.580, *df* = 8, *n* = 42*, P* = 0.227, *CMIN/df* = 1.322, *CFI* = 0.981, *RMSEA* = 0.089), respectively. Comparison with the hypothesized model, the non-significant pathway (*P* > 0.05), the direct effect of ENVIRONMENT on AGC (Environment → AGC) was removed. These final models explained 78%, 67%, and 59% variation of TRAITS for at the 40 m × 40 m, 60 m × 60 m, and 80 m × 80 m scales, respectively, and the explained variations of AGC increased from 29% through 33% to 47%, respectively (Figures 1B–D). The SEMs revealed direct effect of ENVIRONMENT on TRAITS as well as TRAITS on AGC, meanwhile there was no significant direct effect of ENVIRONMENT on AGC. However, ENVIRONMENT had an indirect effect on AGC through its effects on TRAITS. TRAITS in the SEM apparently mediated the direct effects of ENVIRONMENT on AGC.

Terrain convexity always had the highest effect (>0.7) among ENVIRONMENT at each scale (Figures 1B–D). The direct effect of SOM increased slightly (0.45 to 0.57) while that of EB increased greatly (-0.29 to -0.57) with the increase of spatial scales. TRAITS always had a large effect (>0.9) on CWM_SLA and CWM_WD. ENVIRONMENT had a strong negative effect on TRAITS and this direct effect decreased with the increase of spatial scales (-0.89 to -0.77). TRAITS also had a strong negative effect on AGC and this direct effect increased with the increase of spatial scales (-0.54 to -0.68). ENVIRONMENT only have an indirect effect on AGC through TRAITS and this indirect effect expanded gradually with the increase of spatial scales (0.45 to 0.53, in Supplementary Table S2). TRAITS always had a larger effect on AGC than ENVIRONMENT at each spatial scale and the difference of this effect on AGC increased gradually from 40 m × 40 m (0.45 for Environment and -0.54 for Traits) to 80 m × 80 m (0.53 for Environment and -0.68 for Traits).

Overall, TC played the most important role in affecting TRAITS and AGC among environmental variables, meanwhile SOM and EB became more and more important on driving variations of TRAITS and AGC with increasing spatial scale. ENVIRONMENT always had a strong effect on TRAITS, though this effect decreased gradually with the increase of spatial scale. TRAITS also had a large effect on AGC and its effect increased with increasing spatial scale. Therefore, TRAITS seemed to act as the mediators in regulating the effects of environmental change on AGC. Moreover, TRAITS always play a larger role in affecting AGC than environmental factors at each spatial scale.

## Discussion

### The Response of Functional Traits to Environmental Factors

Environmental factors can be considered as filters constraining which individuals bearing specific attributes of ‘response traits’ are able to be sorted and persist in a community (Keddy, 1992). Different sets of response traits to environmental factors have been recognized in plants (Ackerly, 2004; Kraft et al., 2008; Poorter et al., 2009). The response of ‘conservative’ strategy (low specific leaf area and high wood density) to larger TC indicates that there is higher specific leaf area and low wood density in valley than in ridge. The valley habitat is often characterized by high soil water content and nutrient availability (Jobbágy and Jackson, 2003), which always lead to high specific leaf area and low wood density (Baker et al., 2004; Douma et al., 2011). The species in valley have to expand their leaf surface area to gain a higher light availability due to a shaded condition (Andersen et al., 2012). And there is more species living in valley due to a greater light heterogeneity, especially some shrub species, which often have lower wood density than tree species (Bu, 2013). The reason that why TC plays the most important role in affecting functional traits among environmental factors may be attributed to that it is a comprehensive index of environmental heterogeneity (Kusumoto et al., 2013). In general, topographic features such as TC relate to light availability, water shifting and nutrient distribution (John et al., 2007). Unlike soil properties, topography is not a direct environmental variable but an indirect, or a proxy variable that characterizes the overall quality of a habitat (Legendre et al., 2009).

The response of ‘conservative’ strategy to high SOM was in line with the report of Garnier et al. (2004) in grassland. SOM is mostly derived from plant inputs that have undergone processing by microorganisms in the presence of potential reactions with soil minerals (Hobbie et al., 2007). Increases of SOM during the course of succession are well documented (Lavorel et al., 1999), even in old-growth forest with time (Zhou et al., 2006). It is probably due to a combination of an increase in the rate of litter input per unit ground area as the standing biomass of the communities increases, and a decrease in the rate of litter decomposition, as fast growing species that produce high-quality litter are progressively replaced by slow-growing species producing a litter of lower quality (Garnier et al., 2004). In our study, slow-growing species with low specific leaf area and high wood density also had a low rate of litter decomposition and then caused an increase of SOM. Therefore, ‘conservative’ community tends to hold high SOM.

Cation uptake and supply may explain the positive effect of EB on functional traits. Atmospheric inputs of Ca, K, and Mg are usually less than plant uptake, which is typically supplied through mineral weathering, mineralization, and leaching from plant biomass (Berthrong et al., 2009). In our study, atmospheric inputs, mineral weathering, mineralization might be similar across the 30-ha plot of old-growth forest, but leaching from plant biomass will change by different community functional traits. Fast-growing species with high specific leaf area and low wood density generally had a high rate of litter decomposition and nutrients in litter can return quickly to soil (Reich, 2014). Therefore, the change of EB in soil may correlate with the turnover of nutrients and their cycling in plant-soil feedback (Jobbágy and Jackson, 2003). Because plant litter in bark, leaves, twigs, and reproductive structures contained the majority of the total biomass Ca and Mg (Day and Monk, 1977), community with “acquisitive” strategies tend to have higher exchangeable cations through accelerated leaching from plant biomass.

In our SEM, ENVIRONMENT was composed of TC, SOM, and EB. TRAITS composing of high specific leaf area and low wood density were corresponded to ‘acquisitive’ plants with fast nutrient acquisition and turnover. Thus, the negative relation between ENVIRONMENT and TRAITS implies large TC, high SOM and low EB will make community tend to ‘conservative’ strategy (low specific leaf area and high wood density). The tight correlation between variation in habitat preference and plant economics spectrum suggests that the topographically and edaphically associated environmental filter mediated through morphological and physiological traits of species is an important mechanism in shaping spatial patterns of tropical forest community (Kusumoto et al., 2013).

### The Effects of Functional Traits on Aboveground Carbon Stock

Carbon storage in vegetation and soil underpins climate regulation through carbon sequestration. Given continuous plant growth, functional traits that optimize acquisition and efficient use of solar radiation and nutrients throughout the forest complex canopy are very important for sustained ecosystem carbon input (Reich et al., 2014). In our study, the significant negative relationship between CWM_SLA and AGC and the positive relation between CWM_WD and AGC suggested that community composing of high specific leaf area and low wood density plants (‘acquisitive’ strategy) will gain low AGC stock, meanwhile community composing of low specific leaf area and high wood density plants (‘conservative’ strategy) will gain high AGC stock in the old-growth rainforest. Because different plant species differ in their ability to capture, store and release carbon, community functional traits should be a major driver of carbon sequestration in terrestrial ecosystems (De Deyn et al., 2008). The negative relation between TRAITS (high CWM_SLA and low CWM_WD) and AGC in SEM also supports that community composing of ‘acquisitive’ plants will gain low AGC and that composing of ‘conservative’ plants will gain high AGC in old-growth rainforest. The amount of wood in the vegetation and its carbon content determined the total amount of carbon stored in biomass. Plant traits associated with a higher investment in structure per unit of biomass, such as wood density, are therefore expected to directly influence aboveground and belowground ecosystem carbon storage (Baker et al., 2004; Moles et al., 2009). Moreover, there is evidence of a trade-off between a suite of attributes promoting fast carbon acquisition and fast decomposition and another suite of attributes which promote the conservation of resources within well-protected tissues and slow decomposition (Wright et al., 2004; Luoto et al., 2007; Cornwell et al., 2008; Poorter et al., 2009). Therefore, at the ecosystem level, prevailing acquisitive trait syndromes should be conducive to higher carbon fluxes, whereas prevailing conservative trait syndromes should be conducive to higher carbon stocks (De Deyn et al., 2008; Bu, 2013; Conti and Díaz, 2013).

The role of functional traits at community level has been shown for aboveground net primary productivity (Mokany et al., 2008) and carbon-cycling (Bardgett et al., 2013). Consistently, the significant relationships between community functional traits (CWM_SLA and CWM_WD) and AGC were found in the old-growth tropical rainforest. Our result also supports the mass ratio hypothesis in agreement with the report by Garnier et al. (2004). In the mass ratio hypothesis, Grime (1998) suggested that the extent to which the trait of a species affects ecosystem properties strongly depends on the relative contribution to the total biomass of that species. Accordingly, ecosystem functions [e.g., carbon cycling (De Deyn et al., 2008)] would be determined by aggregated traits of dominant species.

### The Indirect Linkage Between Environmental Factors and on Aboveground Carbon Stock Through Functional Traits

Focusing on plant functional traits rather than on species identities, allows generalizing complex community dynamics and predicting effects of ongoing environmental changes (Klumpp and Soussana, 2009). The framework proposed by Suding et al. (2008) describes an approach to scale up from individuals to communities and ecosystems in the context of environmental change. The physical environment can strongly influence variation in ecosystem functions (Boyer et al., 2009; Geert Hiddink et al., 2009; Lavorel et al., 2011), which can be due to both shifts in functional composition along environmental gradients and direct effects of the physical environment (Díaz et al., 2007; Gross et al., 2008). In our study, we expected that environmental factors would have a direct effect on AGC. Contrary to our expectations, the environmental factors had a large effect on community functional traits and only had an indirect effect on AGC through community functional traits in the old-growth forest. The tight correlation between variation in habitat preference and plant economics spectrum suggests that the topographically and edaphically associated environmental filter mediated through physiological traits of species is a vital mechanism shaping spatial patterns of tropical forest community (Kusumoto et al., 2013). These results also indicate that in forest ecosystems, plant functional traits optimize acquisition and efficient use of solar radiation and soil nutrients to sustain ecosystem carbon input and turnover by different plant strategies (“acquisitive” or “conservative” strategies). Therefore, community functional traits always had larger effects on AGC than environmental factors. Michaletz et al. (2014) studied variation in terrestrial net primary production across global climate gradients and concluded that age and biomass of plant (functional traits) explained most of the variation in production whereas temperature and precipitation (environmental factors) explained almost none, suggesting that climate indirectly (not directly) influences production.

We found that although environmental factors always had a strong effect on community functional traits, the effect decreased gradually with the increase of scale size. This could be due to the homogenizing effect of topographic variables at the larger scales (see Supplementary Table S1) and other unaccounted environmental variation at the smaller scales (Punchi-Manage et al., 2013). This observation is consistent with results of other studies of tropical forests: the relationship between community functional traits and habitat heterogeneity is scale-dependent, which suggests that species functional characteristics related to resource exploitation and fitness adaptation can be spatially variable across different scales (Lavorel et al., 1999; Díaz et al., 2007; Hu et al., 2014).

We found that the effect of community functional traits on AGC increased with increasing scale size. On one hand, the sampling unit of 40 m × 40 m may be too small to well reflect the local woody species pool of a site in the species rich tropical rainforest. Thus, random effects introduce noise and impede the detection of ecological patterns, which depend on the spatial resolution (Luoto et al., 2007; Steinmann et al., 2009). On the other hand, the large effect of community functional traits on AGC supports the mass ratio hypothesis, which suggests that the effect of a given species on ecosystem processes is proportional to its relative contribution to the total biomass of the community. Therefore, community-weighted mean trait effects on AGC stock should increase with sampling scale due to additive effect and the effects of community weighted mean functional trait on ecosystem functions should be scale dependent. There are several studies, indicating that the plant community trait effects may primarily be attributed to the biomass ratio hypothesis rather than functional diversity hypothesis (Mokany et al., 2008; Laughlin, 2011; Lavorel et al., 2011).

## Conclusion

Functional traits have a potential to describe how the functional composition of communities responds to environmental gradients and how they influence ecosystem processes and services delivery. Furthermore, the relative effects of environmental changes on ecosystem functioning in a rapidly changing world still needs to be evaluated (Hillebrand and Matthiessen, 2009). To disentangle the relative effects of these factors on the functioning of a species diverse forest ecosystem, we assessed the mechanistic linkages between environmental variations and AGC stock through functional traits across three nested spatial scales by SEM. Our results indicated that stands composed of ‘acquisitive’ species (high specific leaf area and low wood density) gained low AGC meanwhile stands composed of ‘conservative’ species (low specific leaf area and high wood density) gained high AGC. These community level functional traits effects on AGC stock increased with increasing spatial scale in accordance with the biomass ratio hypothesis. Plant functional traits at community level responded to physical environmental variations while affected stand AGC stock directly in each spatial scale. Changes in abiotic site conditions did not directly affect AGC stock significantly, but indirectly via changes in plant functional traits. Plant functional traits are more important than environmental factors in driving variations of stand AGC stock in the tropical montane rainforest. Our study suggests that plant functional traits are the mediators in regulating effects of changes in abiotic site conditions on ecosystem functioning. Although our conclusion is largely supported by the data in the old-growth tropical forest, ecosystem functions are also affected by species richness (Liu et al., 2018), trait diversity and phylogenetic diversity (Cadotte et al., 2009); tree size (Stephenson et al., 2014) and so on.

## Author Contributions

RZ, YL, and YD designed the study. ML, JW, and CZ collected the field samples and performed the plant traits and carbon stock measurements. WB, HX, and JH analyzed the data and performed the statistical analyses. WB and JH wrote the first version of the manuscript, which was intensively discussed and revised by all authors.

## Conflict of Interest Statement

The authors declare that the research was conducted in the absence of any commercial or financial relationships that could be construed as a potential conflict of interest.
