# Supplementary material for: Plant Functional Traits Are the Mediators in Regulating Effects of Abiotic Site Conditions on Aboveground Carbon Stock-Evidence From a 30 ha Tropical Forest Plot
Source: Front Plant Sci. 2019 Jan 9;9:1958. doi: 10.3389/fpls.2018.01958 (PMC6333873; doi:10.3389/fpls.2018.01958)
Supplement: Supplementary file 1 [file Table_1.docx]

**Plant functional traits are the mediators in regulating effects of abiotic site conditions on aboveground carbon stock-evidence from a 30 ha tropical forest plot**

Wensheng Bu^1,2,3^, Jihong Huang^2,3^, Han Xu^4^, Runguo Zang^2,3^, Yi Ding^2,3^, Yide Li^4^, Mingxian Lin ^4^, Jinsong Wang^5^, Cancan Zhang^1^

^1^*2011 Collaborative Innovation Center of Jiangxi Typical Trees Cultivation and Utilization, Jiulianshan National Observation and Research Station of Chinese Forest Ecosystem, College of Forestry,* *Jiangxi Agricultural University,* *Nanchang 330045, China*

*^2^ Key Laboratory of Forest Ecology and Environment of State Forestry Administration, Institute of Forest Ecology, Environment and Protection, Chinese Academy of Forestry, Beijing 100091, China*

*^3^Co-Innovation Center for Sustainable Forestry in Southern China, Nanjing Forestry University, Nanjing 210000, China*

^4^*Research Institute of Tropical Forestry, Chinese Academy of Forestry, Guangzhou 510520, China*

^5^*Key Laboratory of Ecosystem Network Observation and Modeling, Institute of Geographic Sciences and Natural Resources Research, Chinese Academy of Sciences, Beijing 100101, China*

**Corresponding author:** Runguo Zang, Tel.: +86 01062889546;

Fax: +86 01062884972; E-mail: [zangrung@caf.ac.cn](mailto:zangrung@caf.ac.cn)

**SUPPLEMENTARY MATERIAL**

Variables selection

To reduce the complexity of the final SEM, we considered specific leaf area and wood density at community level (CWM_SLA and CWM_WD) as dependent variables, while topographical factors and soil properties as explanatory variables, a redundancy analysis (RDA) was carried out at different spatial scales (40m×40m, 60m×60m and 80m×80m) to reduce redundancy among these explanatory variables. Results of redundancy analysis (Fig.S1) showed that the first RDA axis (RDA1) explained over 40% variation of functional composition of community, whereas the second RDA axis (RDA2) just explained a small variation. CWM_SLA was tightly associated to exchangeable base (EB), whereas CWM_WD was highly related to terrain convexity (TC) and soil organic matter (SOM).


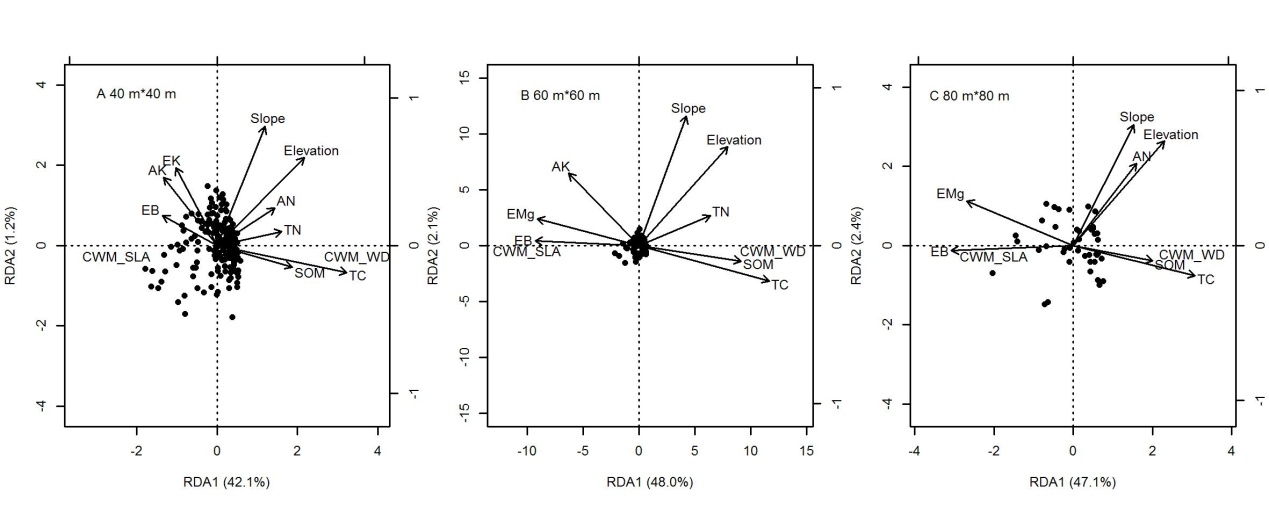


**FIGURE S1** Projections of community functional traits and environmental variables by a redundancy analysis at different spatial scales. Only significant environmental variables are showed (*P<0.05*).

**TABLE S1** Environmental characteristics at different spatial scales (mean±sd).

|  | 40m×40m | 60m×60m | 80m×80m |
| --- | --- | --- | --- |
| Mean elevation (m) | 911.29±24.79 | 911.29±24.44 | 911.85±24.73 |
| Terrain convexity | 0.19±1.86 | 0.19±1.50 | 0.19±1.10 |
| Slope (°) | 24.07±6.01 | 24.07±5.51 | 23.7±5.05 |
| pH | 4.44±0.17 | 4.43±0.13 | 4.43±0.10 |
| Soil organic matter (g/kg) | 49.83±12.53 | 49.75±9.90 | 50.27±7.39 |
| Total nitrogen (g/kg) | 1.98±0.39 | 1.97±0.31 | 1.98±0.25 |
| Total phosphorus (g/kg) | 0.12±0.02 | 0.12±0.01 | 0.12±0.01 |
| Total potassium (g/kg) | 7.12±7.03 | 7.08±6.11 | 6.36±3.61 |
| Available nitrogen (mg/kg) | 158.19±29.3 | 158.09±23.4 | 158.81±19.87 |
| Available phosphorus (mg/kg) | 2.81±0.79 | 2.79±0.56 | 2.8±0.45 |
| Available potassium (mg/kg) | 147.30±35.22 | 145.51±27.35 | 146.17±23.08 |
| Exchangeable potassium (cmol/kg) | 0.39±0.10 | 0.39±0.07 | 0.39±0.06 |
| Exchangeable sodium (cmol/kg) | 0.043±0.013 | 0.042±0.010 | 0.042±0.009 |
| Exchangeable calcium (cmol/kg) | 0.26±0.16 | 0.26±0.13 | 0.27±0.11 |
| Exchangeable magnesium (cmol/kg) | 0.28±0.11 | 0.28±0.08 | 0.28±0.06 |
| Exchangeable base (cmol/kg) | 0.98±0.30 | 0.97±0.23 | 0.97±0.17 |

**TABLE S2** Standardized direct (Dir.) and indirect effects (Ind.) of environmental factors and functional traits. Path coefficients between variables are standardized partial regression coefficients. All effects were significant at *P* < 0.05.

| Scale | Variables | ENVIRONMENT | | TRAITS | |
| --- | --- | --- | --- | --- | --- |
|  |  | Dir. | Ind. | Dir. | Ind. |
|  | TC | 0.7 |  |  |  |
|  | SOM | 0.45 |  |  |  |
|  | EB | -0.29 |  |  |  |
| 40m×40m | CWM_SLA |  | -0.8 | 0.91 |  |
|  | CWM_WD |  | 0.87 | -0.99 |  |
|  | TRAITS | -0.89 |  |  |  |
|  | AGC |  | 0.45 | -0.54 |  |
|  | TC | 0.79 |  |  |  |
|  | SOM | 0.63 |  |  |  |
|  | EB | -0.45 |  |  |  |
| 60m×60m | CWM_SLA |  | -0.75 | 0.92 |  |
|  | CWM_WD |  | 0.81 | -0.99 |  |
|  | TRAITS | -0.82 |  |  |  |
|  | AGC |  | 0.47 | -0.57 |  |
|  | TC | 0.73 |  |  |  |
|  | SOM | 0.57 |  |  |  |
|  | EB | -0.57 |  |  |  |
| 80m×80m | CWM_SLA |  | -0.76 | 0.99 |  |
|  | CWM_WD |  | 0.71 | -0.93 |  |
|  | TRAITS | -0.77 |  |  |  |
|  | AGC |  | 0.53 | -0.68 |  |
